# A Critical Lecture on the Extirpation of the Parotid Gland, and Its Liability to Malignant Diseases

**Published:** 1860-02

**Authors:** Titus Deville

**Affiliations:** Prof. of Anatomy in the Medical Department of the Lind University; Associate of King’s College, London, and late of the Ecole Pratique of Paris etc.


### THE CHICAGO MEDICAL EXAMINER

Vol. I. FEBRUARY, 1860. No. 2

### ORIGINAL COMMUNICATIONS.

*A Critical Lecture on the Extirpation of the Parotid Gland and its liability to malignant diseases.* By Titus Deville, M. D., Prof. of Anatomy in the Medical Department of Lind University; Associate ot King's College, London, and late of the Ecole Pratique of Paris, etc.

*Mr. President and Gentlemen:*

At the last regular meeting of the Chicago Medical Society, Dr. Wickersham, in a paper read by him, spoke of abscess of the parotid gland; to which I replied, by expressing my belief that there was no such disease, and that the salivary glands were almost exempt from morbid structural changes, only one disease being spoken of by sound pathologists, enchondroma, and which has its origin, I believe, in the fibrous tissue, and not in the true structure of the gland. What Dr. Wickersham supposed to be abscess of the parotid gland, was in reality either abscess of the lymphatic glands, one or more, *placed on, or imbedded in the parotid*, or one which had its origin in the areolar tissue. Since that time my attention has been directed to an article in the *Chicago Medical Journal* for this month, December, 1859, written by Dr. Brainard, entitled, "cases of extirpation of the parotid gland and other glandular tumors."

In entering upon this discussion I feel impressed with its serious nature, for I am fully aware of the professional reputation of the surgeon whose authority has been heretofore unquestioned in this section of the country, but I believe it the sacred right and bounden duty of every intelligent man in science to uphold truth and combat error. Those who have seen me either in the lecture room, in medical re-unions, or in private society, know that I vehemently oppose what I feel convinced to be untrue in medical science, and if every now and then in the course of this lecture I am betrayed into an attitude and mode of expression which would appear to many of you, to whom I am unknown, as dictated by rank personal animosity, believe me, seriously, that you are in error, friend or foe I oppose with all my might and strength, so long as I feel assured that truth is supporting me. Whatever I undertake, I put my whole soul into it.

If I prove to your satisfaction that Dr. Brainard is in error, I plead that as an ample justification for a severe criticism, but if not, then I will agree that there are no terms sufficiently forcible for you to express your disapprobation. I would rather have met the discussion in another form, but from the great desire not in any way to implicate my friends, and for the reasons assigned by the President this evening, I felt there was no other course open to me. It would be mean to attack a little man, but a man of great reputation is a fair game, provided you can prove he is in the wrong, or else the crime is proportionately greater. It is by essays like this one of Dr. Brainard, that science is materially retarded. Ought not surgical science, at every turn, to receive an impulse from a man in such a position in the profession, rather than retardation. When such productions are read in Europe by hospital surgeons, what do they think of American surgery? They are led to cast grave doubts on peculiar cases which are reported, and which may nevertheless be perfectly true. The principles and practice of modern surgery must be supported by sober and undeniable facts.

I beg to enter my solemn protest against the deductions of the author as given in the article written by him, and openly avow that he displays in it an ignorance of the teachings of anatomy and sound pathology, that he is professing dangerous and erroneous doctrines in surgery, and advocating them to the profession. These remarks are to be understood as applying only to the subject under discussion, any other I shall not, at this moment, enter upon. I do not commence it for the sake of opposition; I have already strongly protested against these opinions, before I knew anything about Dr.

Brainard's views. Remember, Gentlemen, that we are living in the latter part of 1859. Dr. Brainard is at this time teaching these doctrines and promulgating them through his journal. Now this is a discussion of the very highest surgical importance to myself and to the profession, all I desire from you is to give me a fair hearing; for one of two things must naturally result therefrom, and on which I am content to take issue; either Dr. Brainard from henceforth falls from the high position he has so long held in these western states, or I must be ranked as a bold and ignorant impostor!

Now what are my especial claims to authority on this subject.

1st. I have carefully dissected the parotid region, scores of times, with the view of demonstrating the great difficulty of the *complete* extirpation of the gland, and the extreme danger of wounding parts which are *immediately essential* to the life of an individual.

2d. I have carefully studied the so-called diseases of the parotid, have seen several myself, but on extirpation have invariably found them to be a *diseased lymphatic gland, and not a diseased parotid.* I will admit that there may be cancerous infiltrations and ulceration extending to it from malignant disease in the immediate neighborhood, and also pus found in the ducts of the parotid. To doubt also that the parotid could take on inflammation would be erroneous, but if the elements of the parotid be inflamed, I believe it is by propagation and sympathy, and not as having origin in them.

3d. I have performed, witnessed or assisted in more than 2,000 autopsies, passed weeks in inspecting and studying the pathological collections of the Hunterian Museum of the Royal College of Surgeons, London, and the Musee Dupuytren, Paris. In connection with the Medical Societies of London and Paris I have seen an infinite number of morbid specimens, *but never one of the parotid, strictly so speaking.*

Before addressing myself in detail to the assertions put forth by Dr. Brainard, let me here briefly review; 1st, some of the more important points relating to the surgical anatomy of the parotid gland. 2d, a brief summary of the anatomy of the lymphatic vessels and glands found in the region of the parotid.

*Fascia enveloping the Parotid. Superficial portion.* This is continuous below and behind with the cervical fascia, and is usually an extremely dense layer, not only binding down the gland to the surrounding parts, but also sending septa into its structure, isolating its lobules and adhering closely to them, attached superiorly to the zygoma, anteriorly continuous with the fascia covering the masseter muscle. *Deep portion.* It is connected posteriorly to the mastoid process, the tendinous edge of sterno-mastoid and cartilage of ear; this portion is remarkably dense and so firmly adherent to the gland as to render its dissection very difficult when an attempt is made to extirpate the gland; anteriorly, it passes deeply to be connected with the styloid process and is continuous with the stylo-maxillary ligament, (which separates the parotid from the sub-maxillary gland) and also with the spheno-maxillary ligament; superiorly it is attached to the vaginal process, and thus it forms a pouch for the parotid, but the gland is so firmly attached to its fibrous sac, by means of the processes which separate the lobules, and also prolonged over the parts of the gland which insinuate themselves into the various recesses and interstices of the irregular parotidean cavity, as to render the total enucleation of the gland almost impossible.

The parotid moulded to the walls of the excavation in which it is received is of an irregular form. *External surface, or base* of the gland corresponds to the skin, it is of a somewhat irregular quadrilateral form, but a portion of the gland, '*socia parotidis*' is prolonged forward with the duct over the masseter muscle. The *anterior surface* of the parotid is grooved to receive the posterior border of the ramus of the jaw, and corresponds to the internal pterygoid muscle, stylo-maxillary ligament, and masseter muscle, on the external surface of which it is prolonged for a varying distance, separated from it by some areolar tissue, branches of the portio dura nerve and transverse facial artery. A process of the gland is prolonged between the condyle of the lower jaw and stylo-maxillary ligament, *lying around the internal maxillary artery.* The *posterior surface* of the parotid corresponds to the cartilaginous portion of the external meatus, upon the convexity of which it is moulded, and to which it is connected by dense areolar tissue and also to the mastoid process, sterno-mastoid and digastric muscles. *Superiorly* the parotid is in relation with the zygoma and tempero-maxillary articulation. *Inferiorly* it fills up the space between the angle of the jaw and the anterior border of the sterno-mastoid muscle. It here comes in relation with the sub-maxillary gland, but is separated from it by the stylo-maxillary ligament. The *internal or deep surface* of the parotid is very uneven, it fills up the posterior part of the glenoid cavity and the space between the ear and ramus of the jaw; it surrounds the styloid process and the muscles which arise from it, and passes down between the styloid process and pterygoid muscles, *and is only separated from the pharynx, and internal carotid artery, as well as the internal jugular vein, also the spinal accessory and glosso pharyngial nerves by a comparatively thin layer of areolar tissue, and a little deeper lie the pneumo-gastric, hypoglossal and great sympathetic nerves.* In addition to these relations which it bears to the parts by which it is surrounded and limited, the parotid has important relations to vessels and nerves which pass through its substance. 1st. The external carotid artery traverses the gland from below upwards and on its inner side, giving off in its course the auricular anterior and posterior, with other small branches, and dividing in its substance into the internal maxillary and superficial temporal. 2d. The temporal and internal maxillary veins, which uniting in the substance of the parotid, form the trunk of the external jugular, which is joined by the posterior auricular and transverse facial veins; also *there is a branch of communication between the external and internal jugular veins which traverses the parotid.* 3d. The trunk of the facial nerve is found to divide in its substance into several branches, the superficial temporal of the inferior maxillary nerve traverses it, also the great auricular, but more superficial.

To any one who may be skeptical of this anatomical description, I will demonstrate its truth on the dead subject in the dissecting room of our Medical College.

2d. *Lymphatics.* The lymphatic vessels which course towards the parotid and traverse it, open into the ganglions of the upper part of the neck and come from several sources.

1st. Those from the anterior third or half of the scalp pass vertically to connect themselves with one or two ganglions situated near the summit of the gland.

2d. Those from the eye-brow and the external portion of the eyelids, the skin over the cheek and parotid region open into ganglions situated in the thickness of the parotid, and in general so small that it is difficult to discover them, unless the lymphatic vessels are injected and traced to their termination.

3d. Those lymphatic vessels which come from the external surface of the pinna of the ear converge towards a ganglion in front of the tragus, which is generally very apparent.

4th. Those which come from the helix, and all the internal or posterior surface of the pinna, turn round the external meatus to empty themselves into two or three ganglions of variable size, which are situated on the inferior part of the posterior border of the parotid.

All these ganglions are the *superficial set*, and lie beneath the fascia, but there are others, two or three in number, which are situated deeply behind the external carotid and along its course. They exist constantly and are generally very small. M. Huguier (Gaz. des Hopitaux, 1849, p. 97,) describes ganglions beneath the parotid, between the styloid muscles. These deep ganglions, according to Theile,* receive the deep lymphatics of the face, buccal region, soft palate and its pillars.†

I shall divide my authorities into three classes.

1st. To the most eminent anatomical authorities for the decision as to whether the extirpation of the parotid gland is a practicable, safe and easy operation or not.

2d. To the greatest surgical authorities to decide as to whether they have ever seen a case of diseased parotid, or not.

3d. To the records of pathological museums and the testimony of the most reliable authorities in pathology, are reserved the proofs of the non-existence of malignant disease of the parotid.

---

* Encyclo. Anatomique Allem ; trad : par M. Jourdan.

† I am much indebted to the Encyclopoedia of Anatomy and Physiology, the descriptive Anatomy of M. Sappey, on these points.

The first two lines of Dr. Brainard's paper read thus: "The possibility of removing the whole of the parotid gland by operation is hardly called in question by intelligent surgeons."

In reply, I will not deny the utter impossibility, but I contend that the entire removal of the parotid gland is doubted by the most intelligent anatomists and surgeons, with whom I coincide, and it is an operation which Dr. Brainard would not willingly undertake.

In the first place, I beg to refer you especially to what has been already stated by me, respecting the surgical anatomy of the parotid gland. I submit that I have therein given clear proofs that the *entire removal* of the parotid must be a most dangerous operation and most difficult of execution.

1st. From the form and manner in which the gland is attached to its fibrous envelope, from the extreme irregularity of the gland, and from the many processes which insinuate themselves in the interstices of important parts difficult to reach, rendering its complete enucleation almost impossible.

2d. Recollecting that the external carotid artery and its branches, the external jugular vein and its tributaries, together with the branch of communication between the external and internal jugular veins would almost of necessity be cut in such an operation, the frightful amount of hemorrhage which would ensue, proceeding from the bottom of a deep, narrow and irregular wound, the almost insuperable difficulty of controlling it, would make the boldest surgeon hesitate before attempting such an operation.

3d. Lying immediately beneath the parotid gland, and only separated by a comparatively thin layer of areolar tissue, is the internal jugular vein and internal carotid artery, the eighth and ninth pairs of nerves, together with the grand sympathetic, any injury to which would place the life of the patient in the utmost jeapordy.

Let us quote a few anatomical authorities on this point.

1st. Cyclopœdia of Anatomy and Physiology. Vol. 3, p. 904. Article, "*Parotid region.*" Several lymphatic glands are found imbedded in the superficial surface and in the substance of the parotid. These may readily be distinguished from the tissue of the parotid by their red color.

They are not uncommonly the seat of disease, and if their removal becomes necessary, the operation may be done *without much difficulty and without great risk af wounding any important textures.* But a slight consideration of the deep connections of the parotid and of its close relations to the many important parts which pass through it and by which it is surrounded, will be sufficient to convince the surgeon that the removal of this gland cannot be effected without extreme difficulty and danger, and that it must necessarily be attended by injury to some of the more important parts in this region."

2d. A treatise on Surgical Anatomy of the head and neck, by Allen Burns. (2d Edition, p. 125.)

"The parotid gland is sunk so deep, and is so firmly locked in between the ascending plate of the lower jaw, and the mastoid process, that when it becomes diseased, the patient cannot open his mouth, and from the effect of the fascia, the tumor is flat; *its extirpation is quite out of the question; its impracticability* is proved by reviewing the connections of this gland; whoever has, in situ, injected this gland with mercury, and then, even where it was healthy and free from preternatural adhesions, and limited to its natural size, has tried to cut it out, would be convinced, when he saw the mercury running from innumerable pores, that the gland extends into recesses into which he could not trace it in the living body; if this be true in health, what must it be in disease, where the parts are wedged and niched into every interstice around? Those who assert that they have extirpated the parotid gland, have, I am fully convinced, mistaken that little conglobate gland, which lies imbedded in its substance, and which does sometimes enlarge, producing a tumor resembling a diseased parotid for the parotid itself."

3d. A treatise on Surgical Anatomy, by Abraham Colles, Professor of Anatomy and Surgery to the Royal College of Surgeons, Ireland. (Part 1st, p. 115.)

"*May not the chronic enlargement of some of these lymphatic glands have been mistaken for a schirrus of the parotid itself, and the removal of such by the knife been boasted of as the extirpation of the parotid gland?* When you contemplate the nerves and blood vessels which pass through the substance of this gland, and also the depth to which it sinks, as it is imbedded between the ramus of the lower jaw and the mastoid process of the temporal bone. When you reflect on the very firm and almost inseparable attachment of the gland to these parts, *you will be very tardy in giving credit to the stories of extirpation of a schirrous parotid gland.* The depth to which this gland sinks is such as renders it difficult, on the dead body, to dissect out that portion which lies between the temporal and lower maxillary bones; and this, with the advantages of having the skin previously stripped off, and the view undisturbed by any hemorrhage. When such difficulties occur in the dead, how can we hope to surmount those which must be superadded in the living body? We shall, however, find still stronger objections to this operation than those which arise from these difficulties. We shall find it attended with such unavoidable destruction of important parts as must render the *attempt most certainly fatal.* First, the portio dura of the seventh pair of nerves must necessarily be cut across; a paralysis of this side of the face would be an inevitable consequence of the division of this nerve. The termination of the external carotid, which is yet to give off the temporal and internal maxillary arteries, enters into the lower extremity of this gland. Unless this be tied before the lower part of the gland is raised, *a violent hemorrhage must instantly carry off the patient.* The difficulty of dissecting down to this artery, and then passing a ligature round it, need not be pointed out to any one who reflects that it passes from under the digastric and stylo-hyoid muscles, as it is about to enter into this gland. Some, aware of the danger and difficulty of this part *of their supposed operation*, assert, that they finished the removal of the parotid by tying a ligature round this portion of the gland, and thus causing it to slough away. But, granting for a moment, the practicability of this step, yet it must appear inconceivable how they could dissect out even the upper portion of this gland. For, independently of its position, and the depth to which it sinks between the temporal and lower maxillary bones; independently too of the embarrassments which must attend the hemorrhage from the unavoidable division of many small arteries and large veins in the first steps of the operation, the surgeon has to cut across the trunk of the internal maxillary artery; for this artery passes off from the continued trunk of the carotid completely across the substance of this gland. So that this gland cannot be detached from one half of the ascending ramus of the lower jaw, without the certain destruction of this artery. The end of the divided vessel shrinking in under this bone *cannot afterwards be secured by ligature or by compression.* Should the operator leave behind him any part of the schirrous gland, he must be aware that his operation will be followed by a return of the disease. If to avoid this error, he should dissect at all deeper than the seat of the upper part of this gland, *he will almost inevitably wound the trunk of the internal carotid artery*, which runs anterior to the root of the styloid process, *or of cutting into the internal jugular vein*, which runs immediately behind this process."

4th. The Surgical Anatomy of the Arteries of the human body, by Robert Harrison, Professor of Anatomy and Surgery University of Dublin, Surgeon to Jervis Street, Hospital. (4th Edition, p. 72.)

"First divide the parotid duct and its accompanying arteries and nerves, and raise them, together with the anterior part of the gland, from the masseter muscle and ramus of the jaw; turning this portion of the gland backwards towards the ear, next divide the temporal vessels, and detach the gland at its superior extremity, then separate it from the cartilage of the ear, from the mastoid and digastric muscles, dividing the portio dura nerve; *the circumference of the gland is thus completely loosened*, and now if the student grasp it firmly with a view to twist or tear it out of its situation, *he will find it very difficult to do so; he may even raise the head of the subject from the table, or break the gland, before he can dislodge it from the deep recess into which it extends itself.* He may observe that it fills the glenoid cavity between the capsular ligament of the jaw and meatus auditorius; on drawing it out of this cavity, a process of the gland is seen to pass inside the ramus of the jaw, *with the internal maxillary vein and artery, between the bone and internal lateral ligament*, and to touch the inferior maxillary nerve; this process often swells out between the two pterygoid muscles into a considerable mass, connected like a distinct lobe to the body of the gland, by the narrow neck that passes on the inside of the ramus of the bone. When this has been dissected out of its situation, and the gland drawn towards the neck, a thick portion of it is seen sinking in between the mastoid process of the temporal bone and the angle of the jaw, and resting on the styloid process, around which it is completely folded, *so as to come in contact with the great vessels and nerves at the base of the cranium;* to this part of the gland the student should pay particular attention; if both veins and arteries have been injected, he may perceive *the promixity of the great jugular vein, as well as of the internal carotid artery;* as the gland passes behind the styloid process, *it touches the vein, the eighth and ninth pairs of nerves,* whilst anterior to this process, *it rests on the internal carotid artery and sympathetic nerve;* this portion of the gland is also extended above the stylo-maxillary ligament, and is attached to the internal pterygoid muscle, where it enlarges very considerably; the manner in which this deep lobe of the gland is thus impacted between the styloid and mastoid processes, and again between the styloid process and the angle of the jaw, *explains the difficulty of tearing it out of this situation,* as some authors have advised in the operation of extirpating this gland, in cases of its enlargement and disease. Before the student proceeds with the dissection of the internal maxillary artery, let him again consider the numerous connections of the parotid gland, let him open the anterior or superficial lobe of it, and expose the ramifications of the facial nerve, and the branches of the external carotid, also the large veins which descend from the temple to meet the great trunk of the internal maxillary, which comes from within the ramus of the jaw; let him reflect on the serious injury that must be inflicted in attempting to remove even this part of the gland, which, however, is comparatively easy to that of the deeper portion, and which can only be accomplished with safety in the living subject, by proceeding with the greatest caution among such important parts, an injury of some of which must be almost certainly fatal. When we consider these natural impediments to the extirpation of this gland, and how these may be increased by disease, and when we take into consideration also, *that malignant disease of this gland is very rare, it is impossible not to question the correctness of many of those superficial accounts which are written of the extirpation of this gland as of an ordinary tumor.* Although the parenchyma of the parotid gland is not very subject to malignant disease, yet tumors of this character not unfrequently arise in its cellular tissue, or in some of the lymphatic glands which lie along its inferior border, or which are imbedded in its substance: when a tumor of this nature increases in size, *its pressure will cause the absorption of the parotid, whose situation it will thus come to occupy, and whose form it will resemble.* My own experience, however, will enable me to say, that such a tumor, even when possessed of considerable size, will admit of removal *with less difficulty and danger than the parotid gland, even in its healthy state,* for it will generally be found invested with a capsule, which will enable the operator, when once it has been fully exposed and loosened, to tear it from many of its connections, and thus to dispense with the knife; such tumors, too, are seldom traversed by the facial nerve, or by the external carotid artery or its branches, nor are they so intimately connected to the deep vessels, nerves and muscles of this region as the parotid is, nor as might be previously apprehended."

The learned author then refers to Allen Burns as an authority, giving the same quotation to which I have already drawn your notice.

5th. Traite d'anatomie topographique, ou Anatomie des Regions du corps humain, par Ph: Fred Blandin, Professor of Anatomy, etc., Surgeon to the King. (2d Edition, p. 188.)

"Schirrous tumors of the parotid region may arise from the parotid, but we must avow that they are nearly always situated in the lymphatic ganglions which immediately lie on this gland. The influence of these enlarged lymphatic glands on the parotid is very great and remarkable, they compress and push it inwards, and atrophy it more or less completely, often even the new state which this gland assumes might lead to the belief that it had been extirpated in an operation, but it rests behind untouched. Besides, the anatomy of this region shews us clearly the gravity of such an operation, all the deep parotidean nerves are inevitably destroyed, above all, the facial. There will be of necessity a large number of vessels cut by the extirpation of the parotid, above all, the trunk of the external carotid artery from whence a startling *foudroyante* (thunder-bolt) hemorrhage is to be feared, hemorrhage which demands the special attention of the surgeon, and against which he ought to be ready prepared. To diminish the fear from this hemorrhage, and to obviate the accidents which may occur from the section of the external carotid, Beclard proposed to tie the external carotid below the parotid before commencing the operation of extirpation. This plan ought always to be adopted on principle to prevent a frightful hemorrhage, and give the surgeon time to effect the most difficult ligatures; above all, that of the internal maxillary artery, which plunges beneath the neck of the condyle of the lower jaw. Notwithstanding the ligature of this artery it still continues to bleed from the numerous communications of its anastomotic branches.!'

6th. Traite d'anatomie descriptive, par J. Cruveilhier, Prof. d'anatomie, Paris, (3me Edn., Tome 3me, p. 256) in a foot note, says:

"These relations (of the parotid) prove to us the almost absolute impossibility of the extirpation of the parotid by a cutting instrument."

7th. Traite pratique d'anatomie Medico-Chirugicale, par M. A. Richet, Professeur agrege a la faculte de Medicine de Paris, Chirugien de l' Hopital, St. Louis, etc. etc. (1857, p. 392.)

" *Surgical and Pathological Deductions.* Of late years the question of the complete extirpation of the parotid, when it has become cancerous, has been much discussed. In a thesis of concours in 1841, A. Berard affirmed in the positive, citing for the support of his opinion several observations which appeared to him to establish beyond a doubt, that it had been done. According to this able surgeon, the external carotid artery and facial nerve are necessarily cut, and establishes their section as a criterion whether the extirpation of the parotid has been complete or not. The anatomical description I have already given appears to me to be a strong presumption *against the possibility of this operation*, and taking them into consideration I cannot agree with A. Berard. In effect, I do not think that any one can maintain that the extirpation of the parotid is an easy operation *when practised on the dead body, and even when the gland is in a perfectly healthy state.* All persons who have been tempted and have essayed the operation on the dead body with the view of studying the parotid excavation, avow that it is very long and very difficult to enucleate all its prolongations, which insinuate themselves in the intervals which are left between the muscles, which form the walls, and above all, if one would respect the important neighboring organs. What will it be, when these difficulties are joined to the flow of arterial and venous blood, the movements of the patient, *the pathological softening of the glandular tissue, which always propagates itself, more or less, to the neighboring parts.* All these circumstances, *singularly complicate the operation on the*

*living, which all the ability and coolness of the surgeon cannot charm away.* For the support of these opinions I will cite two facts which appear to me to cut the question better than all reasonings. In the first, I find even in the thesis of A. Berard himself, he gives a case in which Beclard proposed to extirpate the parotid, and who died, happily for the surgeon, some days before the epoch fixed for the operation. M. P. H. Berard dissected the tumor *and found that it sent a prolongation which introduced itself into the internal jugular vein.* Mr. Berard says, 'we may conceive the embarrassment where such a complication existed, and which no one could know beforehand, would throw the surgeon, but is this a reason to always reject the operation.' M. Richet replies, 'No, without doubt it is not a reason to reject it altogether, but it appears to me to be sufficient to cause always hesitation, even in the boldest surgeon, and above all, to cause us to reject in principle the complete extirpation of a diseased parotid. The second case carries equally with it this conviction. I had under my care a patient affected with *an enormous tumor of the parotid region*, who had been under homoeopathic treatment for it, during six months, in the Hospital St. Marguerite. This poor unfortunate suffered most atrocious pain, which left him no repose neither day or night, he could scarcely open the jaw to swallow small quantities of food, he supplicated me every day to operate upon him, as some persons had deemed it practicable, but I took good care not to be tempted, dreading to meet a complication of the kind which occured to Mr. Berard. Some weeks after his entry the patient died in a state of emaciation difficult to imagine. Wishing to do the operation as I should have done it on the living, I proceeded to the extirpation of the parotid, but I was not long in discovering that it was, even here on the dead body an impossible enterprise. The parotid was entirely converted into a half solid substance of creamy-white color, nearly liquid in the centre, it was only with *an unheard of difficulty* that I succeeded in completely enucleating it from its pouch, and even then, when I thought I had removed it all, I found beneath the internal pterygoid, between the styloid muscles, *and all around the internal carotid artery, the pneumogastric and great sympathetic nerves prolongations of the diseased tissue, which I could not detach but with the handle of the scalpel. Certainly it would not have been possible to extirpate it on the living, without wounding these organs*, and I applauded myself in having resisted the supplications of the patient. *For all these reasons, I think, contrary to the opinion of A. Berard, that the extirpation of a degenerated parotid is an impossible operation.* Moreover, it appears that this able surgeon, has without his seeming to know it, felt the difficulty of sustaining the discussion on this ground. for after having given the different proofs in support of his views, (at p. 220,) Berard adds, 'it is not the question to determine whether a cancerous tumor of the *whole parotid* can be extirpated, but only to prove that all the morbid mass may be withdrawn from the cavity which it occupies.' To which Richet replies, 'without doubt that is the true question, and he cannot put it otherwise than to avow, that under its first form it is not susceptible of a satisfactory solution. It does not appear to me admissable, in truth, that any surgeon who should know beforehand that the cancer occupies all the parotid, even until it has comprised the portion which lies on the pharynx, internal carotid artery, internal jugular vein, and the pneumogastric nerve, *would dream to attempt such an operation.* Thus, reduced to its true limits, the question of the extirpation of the parotid is nothing more than an affair of appreciating each particular case, and that is a matter which neither reasoning nor facts can determine beforehand in an absolute manner. Surgical anatomy has incontestably shewn the difficulty, I will say the almost impossibility of extirpation of the whole of the parotid gland on the living, it is then in the hospital clinique where this question can alone be decided. Relative to the observations collected by A. Berard, as cases of the extirpation of the parotid, where the external carotid artery and facial nerve have been conserved, they prove nothing else than this, the knowledge that the operator had penetrated deeply, and extirpated the greater portion of the gland. But far from serving as a rule, such cases are only exceptional, and to sum up in one word, I think that a prudent surgeon in presence of a cancerous tumor of the parotid ought to abstain, if he is not sure that the degeneration not only does not occupy the entire gland, but a portion very restricted and perfectly limited.

The second point in Dr. Brainard's paper to which I call your notice, is contained in the following words, "might not the possibility of removing the whole of the thyroid body, or of the lower jaw, be denied with equal reason."

I contend that the cases are not parallel, the removal of these parts though they would necessarily be attended with a great loss of blood, yet the hemorrhage could be more easily controlled, and there would not be such a liability to wound parts which are important to life. In the extirpation of the thyroid body, the four branches of the superior and inferior thyroid could be tied before commencing the operation, with comparative facility; but supposing the internal jugular vein and internal carotid artery, which are necessarily endangered by the complete extirpation of the parotid, were wounded at the bottom of the parotidean space, I think it would be almost impossible to control the hemorrhage. In the case of the lower jaw, the facial, transverse facial and internal maxillary arteries and veins would be cut across, but it would be in a large open wound, and ligatures could be applied with no great difficulty.

3d. An important admission, to which I call your especial notice, is found in these words: "Some who were forced to admit the removal of the gland, still contend that it has been done rarely, and deny that most of the cases reported as such refer to the gland at all. Velpeau, in his operative surgery, and Beclard in his thesis, seem to cast doubt upon all those cases in which no great hemorrhage occurred, and in which the face was not paralyzed."

You have already heard quoted the eminent anatomical authorities as to the non-practicability of the extirpation of the parotid, I now come to my second class of authorities, those of the most eminent surgeons of modern times, to decide as to whether they have ever seen a case of diseased parotid or not. Their opinions are entitled to the very highest consideration, and are in my belief conclusive.

* 1st. Professor Fergusson, of King's College, my old teacher, says, in his Practical Surgery, p. 412:

"Twenty years ago it was more the custom to speak of tumors of the parotid than it is in the present day, and for my own part *I cannot say that I have seen a single unequivocal case of the kind.* I have seen many swellings in the seat of the parotid, and have removed many with my own hands, but have invariably noticed that these were, to all appearance, developed in a lymphatic gland; when small, the parotid was slightly compressed or perhaps turned aside; and when large, most of it had disappeared."

2d. The late Professor Liston, of University College, (2d American Edition, p. 326.)

"The tumors *over the* parotid, and behind the ramus and angle of the jaw deserve some notice. These, whether enlargements of the lymphatic glands, or adventitious formations, are bound down by a strong condensed cellular sheath or fascia, and also by the fibres of the platysma-myoides which pass upon the side of the face. This growth is equally extensive among the deep-seated parts, as it is prominent externally. The parotid gland is displaced and absorbed; the diseased mass is imbedded in its substance, and ultimately occupies its place. The vascular supply is abundant, and the nerves become intimately attached to the posterior surface of the condensed cellular cyst. The tumor is firmly fixed in all ways, by its strong investments and firm adhesions, and by its being, as it were, dove-tailed by its processes between the bones. Sometimes, after the removal of tumors of long standing in this situation, I have often exposed the whole cavity betwixt the mastoid process and the ramus of the jaw, the styloid and pterygoid processes, muscles, etc."

3d. Druitt, the best English commentator on Surgery, in his Surgeons' Vade Mecum, (7th Edition, p. 475,) says, speaking of parotid tumors:

"This name may be assigned to those tumors which occur in front of the ear, *over the parotid gland.* Cysts of various sorts, filled with glairy matter or with blood; enchondromatous tumors, pure, or mixed with newly developed gland tissue and enlarged lymphatic glands, are the commonest; cancer may also be met with. Such tumors may of course involve the facial nerve; the facial artery, or the external carotid, or may extend inwards to the pterygoid and styloid processes. 'If there be reason to suspect' says Mr. Liston, 'that the disease is of a malignant nature, and not thoroughly limited by a cellular cyst, no interference is admissable. If, on the contrary, it be at all moveable, has advanced slowly, possesses a smooth surface and is firm, (neither of stony hardness, nor pulpy,) then an operation may

* Dr. Brainard has very ingeniously introduced the name of Prof. Fergusson in his article, which might lead an unwary reader to the inference that this distinguished operator lent the sanction of his high authority to such proceedings. After speaking of the division of the facial nerve, in connection with the extirpation of the parotid, he writes thus: "Mr. Fergusson says that in a case in which he divided the facial nerve, the paralysis became after a time, much less conspicuous than at first."

be contemplated.' If slowness of growth and capability of being *moved freely* concur, the surgeon should remove such tumors; keeping his knife close to the tumor, especially at its deep part, so that it may not divide the nerve or artery, if possible. Sometimes, however, they may be so involved that their division is unavoidable. The patient should always therefore be warned of the possibility of facial paralysis after the removal of one of these tumors."

4th. Velpeau's Operative Surgery, (Amer. Edition, p. 15,) speaking of parotid tumors, he says:

"I will say only in anticipation, that those tumors formed by the parotid gland, of which so much has been said, *have all of them*, or almost all of them, for their basis or point of departure *the lymphatic ganglions* properly so called. It is from having frequently ascertained the truth of this position that I take the liberty at the present time *to affirm it positively*."

Again, (at page 446,) he cites fourteen cases of supposed extirpation, all at that time known to him in England, Germany, France, etc., he sums them up thus:

"*Appreciation of the facts.* The question whether we may or may not extirpate the parotid gland in its totality, appears to me to have been incorrectly stated. The salivary glands, including with them the parotid, scarcely ever degenerate. The tumors that have been removed under their name almost all belong to other tissues and to other organs. Even in the substance of the parotid itself there are a great number of lymphatic ganglions. These ganglions when they swell become fungous, tuberculous and cancerous, and are transformed into bosselated tumors, which spread out, flatten, and disorganize the glandular tissue, and lead to misconceptions of the real charact r of the parts which are extirpated. I have performed extirpations of this kind at least twenty times, to such extent *as to lay bare the whole parotid cavity*, and to be afterwards under the necessity of submitting the tumor to a careful dissection, in order to satisfy myself that the ganglions, rather than the glands, had been the source of the disease. I have, moreover *encountered in this region* meliceromatous, lipomatous, fibrous, melanotic, encephaloid, and other tumors."

I could go on citing eminent authorities, amongst others of which I name the Professors of the University of Edinburgh, but enough has been said to prove my assertion.

And now, Gentlemen, let me adduce, if it be possible, still stronger proofs *and which appear to me to be unanswerable;* my third class of evidence, viz: the records of pathological museums, and the testimony of the most reliable authorities in pathology, and these are of the utmost value.

1st. Because the records of museums have been collected by generations of the most eminent surgeons and pathologists.

2d. Because pathologists have not only the light of their own experience to guide their judgments, but those of the specimens to be found in all the great museums of Europe, and the reports of all well authenticated pathological observations.

1st. Manuel d'anatomic pathologique generale et appliquee, contenant la description et la Catalogue du Musee Dupuytren, par Ch: Houel, Conservateur du Musee, etc., etc., (1857) at page 668, speaking of enchondroma, he says: "these pathological productions may arise in the hard or soft parts, we find they arise *in the areolar tissue*, we meet with them particularly in the glands, and they are found in the parotid, the testicle and the mamma, but the most common is in the region parotidean, and often even the cartilaginous mass is contained in the middle of a fibrous mass, more or less considerable."—(Vide preparation No. 23, Musee Dupuy: presented by M. Nealton, which shows well this condition.) A case is reported by M. Richet, (Soc. de Chirurgie Bull. Tome v., p. 88) where a patient died twelve days after the operation on a cartilaginous tumor of the breast, the lungs were found to contain many small cartilaginous tumors, varying in volume from that of a pea to a nut, and even beyond. Mr. Broca, in the discussion which followed, sought to establish the possible hereditary nature of enchondroma, citing in proof a case reported by M. Paget, the specimen of which is conserved in the Museum of the Norfolk and Norwich Hospital."

You see, Gentlemen, the existence of enchondroma is here denied as having origin in the glandular substance of the parotid, and they may be found in any part where fibrous tissue abounds. Is there any other disease whatever of the parotid gland spoken of in this work. *Not a single word.* Let us turn to the Catalogue of the Muséum Dupuytren, and at page 778 what do we find.

Preparation No. 23, to which allusion has already been made. Enchondroma of the parotid, one specimen only. (Nealton.)

No. 24. Tumor, probably hypertrophy of the parotid.
Model in Wax!

No. 25. Cancerous tumor of the parotid.
Model in Wax! (Ruffin.) Italian!

No. 26. Cancerous tumor of the parotid.
Model in Wax! (Sabatier.) Italian!

Is there any other preparation of diseased parotid. Not one!

How comes it that some surgeons in Paris speak of cancer of the parotid, and not a real one to be found in their great pathological museum? The inference is that they were mistaken in their real nature, that they were connected with the lymphatic glands, and on being referred to Robin, their eminent pathologist and microscopist, were rejected. It is the custom in Paris to refer all tumors to his inspection. The older professors have ignored the use of the microscope, and it is only quite recently that Mr. Sappey has placed in the Musee Orfila, microscopical preparations. This anomaly will soon cease to exist before the 'jeune ecole' of France, as it is called, which numbers men of the most enlightened and cultivated talents in pathology, such as Lebert of Zurich, Robin, Broca, Verneuil, Houel, Follin, etc., all of whom are determined at every opportunity to shed light on pathological anatomy, by microscopical investigations.

2d. Catalogue of the Museum of Wm. Hunter.

Not one word of any preparation of diseased parotid whatever.

3d. Pathological Anatomy, by Dr. Wilks, in illustration of pathological collection of Guy's Hospital, London, 1859, at p. 261.

"The salivary glands come next in my list, but I have not many morbid specimens of these to show you. Here is a specimen of calculus, (No $1784^{10}$ in the museum) from the sub-maxillary gland, the obstruction of the duct of which you know is one cause of ranula. From the same cause in Stenos' duct you may have salivary fistula, all of which affections you will hear of from the surgeon. Amongst the new growths affecting these glands is the fibro-enchondroma of the parotid, (No. $1784^{25}$ in the museum.) *One* specimen only. I do not think it is clearly made out where such disease first begins, although the glands are more or less involved. I have already alluded to this form of tumor in my first lecture."

This is the end of diseases of salivary glands, and not another word is said.

4th. Principles and Practice of Medicine, by Prof. J. Hughes Bennett, Edinburgh, 1859. (p. 200.)

"Cartilagenous growths were first described by Muller, under the name enchondroma. In the soft parts they *are surrounded by an envelope of cellular tissue,* and in the bones by a bony capsule. In the first case, they occur, *although very rarely, in the glands,* as in the parotid or mamma. In the second case they are most common in the bones of the extremities. The tumors may be round and smooth, or rough and nodulated from several of them being accumulated together. Though hard to the feel, they often present a peculiar elasticity. They crunch when cut with the knife, usually present a smooth, glistening surface, and are not unfrequently more or less soft, pulpy, gelatinous, and even diffluent in some parts of their substance."

Again, at p. 202, "Enchondromatous tumors are continually mistaken for cancerous growths, a fact pointed out by Muller."

5th. *Paget's Surgical Pathology.* Mr. Paget was called by the council of the Royal College of Surgeons, London, to give a course of lectures in illustration of the pathological collection of the Hunterian Museum, the largest in the world. He not only drew attention in this course to the specimens contained therein, but also to those of other large museums, more especially to that of St. Bartholomew's Hospital, with the Medical School of which he is connected as Professor. He also refers to all the most reliable authorities in Europe. Let us now quote him. (p. 37, vol. 2.)

*Serous Cysts.* "In situation, too, they are various. In some cases they lie in front of the neck; in others; at one or both sides: they may lie by the lower jaw, *over the parotid*, by the clavicle, or anywhere or everywhere in the mid-spaces. And in any of these situations, they may extend very deeply, among the structures of the neck, and may adhere to them so closely, and may so thinly cover them as scarcely to conceal them when laid open. Their date of origin is very obscure. In many, perhaps in the majority of cases, they appear to be congenital; but they may be first observed at any later period of life. Last year, Mr. Lawrence removed a collection of four large cysts *from over the parotid gland and mastoid region* of a man, 28 years old, who had observed their beginning only seven years previously. Three of these were filled with serum, and one with pus."

Again, P. 49, Vol. 2. "In the parotid gland also, cysts containing fluid blood have peculiar interest. In 1848, I assisted Mr. Stanley in the removal of one which lay quite within the parotid of a gentleman about 40 years old. It had been for some years increasing in size, and lay beneath some branches of the facial nerve, from which the need of separating without injury made its removal very difficult. This, however, was safely accomplished, and the patient remains well. At nearly the same time, a man, 25 years old, was under my care with a similar cyst, which had been increasing without pain for two years. It lay in the parotid, but very near its surface. I punctured it, and evacuated two or three drachms of bloody-looking fluid, with some grumous and flocculent paler substance intermingled. This fluid coagulated like blood, and contained blood-cells, much free granular matter, crystals of cholestearine, and what appeared to be white corpuscles of blood acquiring the character of granule-cells. The cyst filled again with similar fluid after being thus evacuated. I therefore dissected it *from the parotid gland*, and the patient recovered."

At p. 201, vol. 2, of the same author.

Cartilaginous tumors *over the parotid.* "The only remaining instances of cartilaginous tumors to which I shall refer are those that grow *near* the parotid, or much more rarely, *near the* sub-maxillary gland." Again, p. 204. "Such are the most general character of these cells; *but they are apt to vary from them*, being more angular, or bearing processes, or being attenuated or caudate. *Even, if we may consider them as imitating gland-structures,* yet it may be a question whether they are related to the adjacent parotid, or to *lymphatic gland.* It would be easy to discriminate between the elements of the parotid and of a lymphatic in their natural state; but a morbid imitation of either of them may deviate far enough to be as much like the other. And it is well to remember that these tumors have *exactly the seats* of naturally *existing lymphatic glands, and are often closely imitated by mere enlargements of these glands;* so that, possibly, future researches may prove that they are cartilaginous tumors *growing in and with a lymphatic gland,* over or *within the parotid,* or sub-maxillary gland."

P. 397. "A firm medullary tumor was *seated deep in the substance of a* young woman's parotid gland. Its removal with the knife *could not be safely completed; about a fourth part of it was left behind,* and the wound was left to heal in the ordinary manner. It healed quickly, enclosing the remains of the tumor; but after some time all the appearance of swelling subsided, and no renewed growth ensued till after a lapse of three months, when it was renewed, but not more rapidly than before."

Mr. Paget, you see, as I have already told you, says not one word in the whole of his work on diseases of the parotid gland, even enchondroma, which I admitted he throws very great doubt upon, and calls them *cartilaginous tumors over the parotid.* Mons. Houel, denies it "in toto," and says it has its origin in the areolar tissue. With the extensive means of illustration at Mr. Paget's command, he does not bring forward one case of malignant disease of the parotid. If neither museums, nor the soundest pathologists can furnish evidence of its disease, what rational conclusion can you come to, *otherwise than that they have no existence.*

In former editions of Dr. Druitt's "Surgeons Vade Mecum," he speaks of malignant diseases of the parotid, but in the later ones he carefully abstains from it, by calling them *diseases over the parotid.* It may be asked, why have I quoted some anatomists who belong to the past generation, and not any of the older pathologists, some of whom have an undoubted reputation? The reason is clear, simple and obvious. Since the days of Blandin and Colles, surgical anatomy has made *comparatively* but little progress, whilst pathological anatomy has made more rapid and brilliant strides than any other branch of medical science. To the microscope we are much indebted for clearing up the doubts which have so long existed as to the nature of morbid growths, by demonstrating their elementary structure. Of the works in pathology that I have quoted, the oldest is Paget, beyond which it would not be safe, in the present day, to refer as an authority.

4th point to which I beg to draw your attention is the following remark by Dr. Brainard: "M. Malgaigne, in his report to the Imperial Academy of Medicine, Oct. 26th, 1858, admits that in certain exceptional cases, on account of anomalies shown by dissections, the parotid gland may be completely removed without wounding the external carotid artery or the trunk of the facial nerve. This conclusion was sustained by the Academy. M. Naegale, as quoted by Velpeau, affirms that in the dead subject the gland may be dissected away without wounding the trunk of the nerve, and that he has removed it in the living without causing paralysis." In reply, I will admit that there are *in rare cases* some differences from the normal relation which the gland bears to the external carotid artery and facial nerve, but is any surgeon to take them into consideration when proposing to operate in this region? Are they frequent? For my own part I never met with such anomalies, and Mr. Malgaigne, (Traite d'anatomie Chirugicale, 2me Edn., Tome 1er, p. 799,) admits that Mr. Sappey, the most distinguished French anatomist, never met with a case in which *the external did not pass through the substance of the parotid.* The Academy of Medicine could also sustain with equal truth, that the external carotid itself *has been known to be entirely wanting.*

5th point. Dr. Brainard next refers to a case of cartilaginous exostosis, published by him in the *American Journal of Medical Sciences*, Oct., 1853, and the entire removal of the parotid "there is no room for doubt." On reading the paper, the only reference to the parotid is given thus: "The parotid gland had been *partially obliterated by pressure*, and no trace of it now remained."

He admits then, he did not remove it; at least, that is the conclusion I draw.

6th point. He says, "If A. Cooper, Beclard, Larrey, Warren, Mott, McClellan, and the scores of surgeons who, knowing the controversy on the subject, have reported operations of the kind, are not to be credited, it will be difficult to find any reliable authority in surgery." In reply, I would say, that if all the most distinguished anatomists, pathologists, and the most eminent surgeons of modern times, whom I have quoted, knowing the controversy on the subject, have denied that they have ever met with any disease of the parotid, (excepting enchondroma) and thrown grave doubts on the so-called extirpation of the parotid, *are not to be credited, it will be difficult to find any reliable authority in surgery and pathology.*

7th point. He says he can report above fifty operations of what he deems undoubted cases of removal of the parotid gland. He also states that he has operated upon five himself.

You have heard me quote Fergusson, Liston, and Velpeau, on this point, who affirm that they have *never met with a single case of a diseased parotid.* Now Gentlemen, seriously and solemnly, do you believe Dr. Brainard before the united and unequivocal testimony of these, the three most eminent operating surgeons of modern times, *if so, there is no other alternative than to admit that Dr. Brainard is the greatest surgical genius, not only of the present, but of every age and country.*

8th point. He writes, "I have added figures of some of the tumors, taken by daguerreotype, believing they would be useful in aiding the diagnosis, for I have generally noticed that each organ in its morbid growth assumes a form peculiar to itself, and believe that careful attention will enable the surgeon to distinguish between enlargements of the parotid and those of the glands situated in contact with it, *by the form and situation alone.*"

It may serve to point out *the difference in situation of a tumor*, but it is an absurd error to insist upon it as an aid to diagnosis between a diseased parotid and lymphatic, and more especially as the existence of the disease in the parotid has been strongly denied by the most competent authorities.

9th point. Description of his two cases of extirpation of the parotid.

Case 1.—*Removal of the Parotid Gland for Scirrhus—Cure.*—Rebecca Dearsdorff, aged thirty years, of full habit and good health, consulted me, January 30, 1857, on account of a tumor situated between the ramus of the jaw and the lobe of the ear. The attention of the patient was first directed to this tumor four years previously, when only a slight enlargement existed. From that time it increased slowly and without any pain until within three months, when it has grown rapidly, and now presents the appearance well represented in the foregoing figure. The Tumor at this time is firm to the touch and presents some inequalities; the skin over it is not discolored.

*Operation.*—The patient having been placed under the influence of chloroform, a semi-circular incision was made, commencing behind the lobe of the ear, ending upon the middle of the cheek, and passing over the lower part of the gland. The covering having been dissected up from below, the finger was thrust beneath the tumor, by which, with an occasional touch with the knife, it was readily separated from its attachments.

A circumstance especially deserving of notice is, that *the fissure in which the gland is naturally situated was nearly empty, and the finger passed into it without difficulty.*

During the operation, the portio dura was divided; the external carotid artery was torn across, and the end of it lay in the lower part of the wound, three-fourths of an inch in length, where it had been drawn out of the tumor. It did not bleed, but as a precaution, it was tied. The face was instantly drawn to the opposite side on the division of the nerve.

On examination of the cavity, the ramus of the jaw, the mastoid process, the styloid process in its whole length, the stylo maxillary ligament, the auditory passage, and the ligament of the temporo-maxillary articulation, were fully exposed. At the bottom, the internal carotid artery and the internal jugular vein were distinctly seen and felt. There was no great hemorrhage. The wound was filled with a sponge until this had entirely ceased, when careful search was made by the surgeons and assistants for any remains of the parotid gland. None could be found; all the spaces into which it is said to prolong itself were vacant. The wound was dressed in the usual way, and a full dose of morphine administered.

On examining the tumor, the structure of a salivary gland was distinctly to be discerned on its entire surface. The trunk of the portio dura passed through it. The central part seemed to me very decidedly scirrhus.

The operation did not occupy ten minutes, and was by no means very difficult. The reason is that during its growth the disease had gradually raised the gland from its bed in a manner which will be readily understood. The patient recovered, and was able to return home in two weeks.

Case 2.—S. S. Millard, aged thirty-five years, consulted me, Nov. 10, 1858, on account of a tumor situated below the left ear. He stated that he had first perceived it about two years previously, and some months since an attempt to extirpate had been made, but the surgeon, finding it deeper and more difficult to remove than he anticipated, desisted, after having cut deeply into it, and contented himself with applying cupping glasses over its surface, by which a small part of its contents had been forced out. The wound cicatrized slowly, and at the time of examination, an irregular surface was presented, and elastic, free from tenderness and pain. The general health was pretty good.

This tumor was removed, Nov. 13, 1858, at the U. S. Marine Hospital, in presence of the class and attendants. Owing to the cicatrix upon the surface, a piece of the skin was removed. I succeeded in getting under it at the lower part and raised it out of its bed by the fingers. There was considerable hemorrhage. The external carotid, the temporal and external maxillary arteries requiring ligatures; the two latter on account of the retrograde circulation. The pharynx, internal carotid artery, internal jugular vein, the pterygoid muscles, were distinctly felt and seen.

The patient recovered without accident, and, Oct. 16, 1859, writes that "my face is apparently well."

On examination of the tumor, it was found to be of a marrow-like appearance, with masses firmer and apparently more fibrous than the rest. The facial nerve passed through it. (The side of the face was paralyzed after the operation.) Surrounding the morbid growth, small portions of the parotid gland, in a healthy state were noticed.

The character of the growth would have left me in doubt as to the malignancy; but the cicatrization after the first incision, and it having remained well for eleven months, leads me to think it was not cancerous.

In this case, the lymphatic glands removed with the tumor were healthy, and the disease seemed evidently to have originated in the parotid. I therefore took great pains to search for any parts which might have remained, and a small piece upon the side of the face was detected and removed after the principal growth had been taken out."

*Comments on Case 1st.*—No good surgeon, anatomist and pathologist will believe that in this case the parotid was implicated and that he extirpated it. Does he really believe it himself, or is he imposing on the credulity of the profession? If so, it is an insult to their intelligence. "With an occasional touch of the knife, it was readily separated from its attachments, there was no great hemorrhage, the operation did not occupy ten minutes, and was by no means very difficult." You have heard the unanimous testimony of all the great anatomists whom I have quoted on this point; every one of them are also distinguished surgeons, either living or dead. How emphatically they have insisted on the great danger, difficulty and violent hemorrhage from extirpation of the parotid. Now do you believe Dr. Brainard in opposition to their united experience? He admits that Velpeau and Beclard cast doubt *on all cases* in which no great hemorrhage occurred; likewise that Beclard and Lisfranc lost their patients by such an operation. Speaking of the removal of the parotid, Dr. Brainard says that Hiester, "describes an operation in which a pound of blood is lost during the incisions, in which death often occurred, and that he criticises Gurengeot who had spoken of it as not dangerous." Dr. Brainard adduces this as a proof that Heister really removed the parotid.

In describing his case, Dr. Brainard says, "the external carotid *lay in the lower part of the wound, for about three quarters of an inch in length*, where it had been drawn out of the tumor. I doubt very much that an artery of the calibre of the external carotid can be severed, and lie denuded for three quarters of an inch *under the circumstances stated, without the slightest hemorrhage*, for the patient was not in a state of syncope, nor was the artery retracted in its sheath, or beneath the fascia.

A diseased parotid, according to Dr. Brainard, is much easier to dissect out than a healthy one! He gives as a reason that the disease had gradually raised the gland from its bed! No doubt an enlarged lymphatic gland in the parotid region, if not very voluminous, is far easier to extirpate than a healthy parotid, but I must protest against the absurd error, that a diseased parotid, supposing it to exist, would be more facile to dislodge than a sound one. A mere tyro in surgery would waver before admitting such an assertion.

He draws particular attention to the following remark: "the fissure in which the gland is naturally situated was *nearly* empty, and the finger passed into it without difficulty." This proves nothing, as all surgeons have insisted that tumors occupying the parotid region, cause the absorption of the parotid and came to occupy its natural seat.

Again, he says, "on examining the tumor the structure of a salivary gland was distinctly to be discerned on its entire surface." Admitting it to be the salivary gland which was seen on the surface, might it not have been a tumor of a deep lymphatic gland, pushing *forward* the salivary gland, which was in a healthy state.

*Comments on second Case.*—The same remarks will apply to the second as to the first, with the exception of the hemorrhage which appears to have been considerable. He says, "I succeeded in getting under it at the lower part, and raised it out of its bed by the fingers." Could he have done so had it been the parotid? He admits that it was a most easy operation. No microscopical examination was made of the tumors. It is a very meagre, loose and faulty description, no surgeon reading it would suppose that it was a case reported by a professor of surgery, and there is no conclusive evidence whatever that the parotid was at all implicated.

*Comment on case* 4.—Enlargement of the Lymphatic Glands in the Parotid Region.

"After the operation was finished, the parotid gland was found in its natural situation, *but so much scooped out* that it was perhaps *possible to have supposed that part of it had been removed.*" Here is an important admission by Dr. Brainard, viz: that enlargement of lymphatic glands may cause absorption and take the place of the parotid. Now, was it not so in the other two cases?

*Analysis of facts and summary.* To support my argument that the *entire removal* of the parotid is a most dangerous operation, and attended with the greatest difficulties, in opposition to that of Dr. Brainard, who contends that it is an easy, safe operation, and one which is not unfrequently attended with but little loss of blood, I have given you my own deductions from dissections, and quoted as anatomical and surgical authorities, the Cyclopœdia of Anatomy and Physiology, Burns, Colles, Harrison, Blandin, Cruveilheir, and Richet.

To support my argument as to whether the most eminent surgical authorities of modern times, *have ever seen a case of diseased parotid*, in opposition to Dr. Brainard, who asserts that he can report above fifty operations of what he deems undoubted cases of removal of the parotid, and has operated upon five himself, I have quoted Fergusson, Liston and Velpeau.

To support my argument as to whether the parotid gland is subject to malignant disease or not, in opposition to Dr. Brainard, who teaches that doctrine, I have quoted the records of the Musee Dupuytren, catalogue of the Museum of Wm. Hunter, and also the unmistakable evidence of the most eminent pathologists, such as Houel, Wilks, Bennett, and Paget.

What is the use of extirpating the parotid, if it does not take on disease? (always reserving enchondroma, and which is spoken of as rare.) From all these considerations, I contend that I have proved beyond the possibility of a doubt, that Dr. Brainard, in the two cases he describes, did not extirpate the parotid; what he thought was the parotid was only a tumor *placed over the parotid, or occupying its place*, and from the unanimous testimony of the anatomical and surgical authorities that I have quoted, prove that such tumors are usually easy to remove, from being limited by a cellular capsule, from which they ordinarily can be enucleated with facility, and that they may or may not implicate the external carotid artery and facial nerve. I also maintain earnestly the justification of this criticism, inasmuch as I think the proofs are irrefragable that Dr.

Brainard is, *on this subject*, in ignorance of the teachings of anatomy and sound pathology, that he is professing dangerous and erroneous doctrines in surgery, and advocating them to the profession.

